# Extracurricular Pulse Activities in School: Students’ Attitudes and Experiences

**DOI:** 10.3390/ijerph192215051

**Published:** 2022-11-16

**Authors:** Veronica Jägerbrink, Joakim Glaser, Anna Hafsteinsson Östenberg

**Affiliations:** 1 Department of Sport Science, Malmö University, Nordenskiöldsgatan 10, 211 19 Malmoe, Sweden; 2 Department of Sport Science, Linnaeus University, 352 95 Växjö-Kalmar, Sweden; 3 Department of Society Culture and Identity, Malmö University, 205 06 Malmoe, Sweden

**Keywords:** academic achievement, health, physical activity, school, students’ attitudes

## Abstract

Background: Few children and adolescents reach the recommended levels of daily physical activity, which is something that affects their health and wellbeing. Research shows that physical activities could be one factor for improving health and achieving academic goals in children and adolescents. Methods: Eight focus group interviews with students 10–15 years old were conducted at two schools with extracurricular pulse activities (ECPAs) during the school day. Results: In general, the interviewed students at both schools expressed positive attitudes toward ECPAs, emphasizing a felt correlation with physical activities out of school. Phenomena such as motivation, concentration and social relations also seem to profit from ECPAs. However, some students display a critical approach to ECPAs. From a gender perspective, girls embrace ECPAs with more enthusiasm than boys. Conclusions: In order to make the best use of positive attitudes and health promotion, schools need to improve structural conditions such as facilities, time pressure, unhygienic conditions, blurry boundaries between ECPAs and Physical Education (PE), uncomprehending teachers, contents and, very importantly, the inclusion of students in the process of planning and implementing ECPAs.

## 1. Introduction

Since habits are established during childhood, interventions such as increased physical activity and information about physical activity to children and their parents seem to be successful factors for achieving these habits [1,2,3,4,5,6]. Physical activities in school have been studied and shown to be important factors when it comes to wellbeing and health [7,8]. However, most young people do not reach the recommended levels of daily physical activity [8,9,10,11]. For example, in Sweden, only 22% of the girls and 44% of the boys in the range of 11–17 years old meet the recommendations of daily activities [11]. It is concluded that more active Physical Education (PE) lessons in school are needed [12], and that the school setting may be the best way to reach young people and to increase the health-related fitness of children [3,4,5,6,13]. The recommended level of physical activity is 60 min of moderate-to-vigorous intensity per day, including three days a week of vigorous-intensity aerobic activities that strengthen muscle and bone [14].

Studies have also shown that physical activities could be one factor for achieving academic goals and increasing the general wellbeing of adolescents [3,6,7,8,15,16,17,18,19,20,21,22,23,24,25]. Additionally, authors have shown that a regular physical activity over several weeks has positive effects on the executive functions and academic performance of preadolescent children [24,25].

In addition, being physically active during childhood is found to decrease the risk of developing chronic diseases later in life [26], prepare children for an active and sustainable life with a minimum of injuries [1,27] and to increase the chances of a physical and active life in the long term [20].

Lindgren found that students perceived an improvement regarding academic performance, health and general wellbeing when engaging in moderate-to-vigorous physical activity three times a week in a school setting [28]. They also experienced that physical activity helped them to better structure their school day. This is in line with both Seger [29], who found that a teacher-led school intervention generated a sustainable project with improvements in physical fitness and school grades, and Molcho [30], who highlighted the need to encourage and enable adolescents, and especially girls, to participate in vigorous exercising as a way of promoting positive mental health.

Since habits are established during childhood, interventions such as increased physical activity and information about physical activity to children and their parents seem to be successful factors for achieving habits of physical activities [1,2,6]. Moreover, research on students’ opinion on extracurricular physical activities (ECPAs) has shown that students experienced positive effects on memory, concentration, motivation and alertness on lessons following ECPAs [7,8,13,20,28,29,30].

Thus, research shows that physical activity has a positive impact on students in and out of school, which is one explanation for the interest in ECPAs over the recent years [6,24,28,29]. The students’ own perspective in this context is an important issue, with reference to students as not one homogenous collective, but as a heterogenous group characterized by different attitudes due to their different experiences and identities. Thus, more studies on students’ experiences of and opinions on ECPAs are needed.

The objective of this study is to increase the knowledge of students’ individual and differing experiences of and attitudes toward ECPAs. More specifically, we are interested in attitudes, possible impact on school, wellbeing and health. Furthermore, it may be possible to gain knowledge on why and for what reasons students have positive or negative attitudes towards ECPAs. Emphasizing the students’ perspective could also deepen the understanding not only of the possibilities and challenges of ECPAs, but also offer a better understanding of how students, teachers and other school staff could approach and structure ECPAs.

## 2. Material and Methods

### 2.1. Participants

The participants in the study were students at two municipal schools in two cities in Sweden who have participated in mandatory ECPAs with the aim of improving both their physical and mental health as well as improving their academic goals. At School 1, the activities involved grade 7–9 students (13–15 years old), whereas at School 2 the activities involved grade 4–6 students (10–12 years old). For this study, students that had participated in ECPAs for nearly two school years were chosen for interviews. Overall, we interviewed 20 students in grade eight at School 1, twelve students in grade six and twelve students in grade five at School 2. At School 1, the interviews were carried out on site, while at School 2 they were conducted via ZOOM due to COVID-19 restrictions.

### 2.2. Procedures

At both schools, we were invited to do research into already existing ECPA projects for a few years. Thus, we had no influence on the design of the two different projects. At School 1, ECPAs were conducted for 20 min three times every week, and at School 2 they were conducted for 30 min twice a week. At both schools, the intention with the ECPAs was for the students to reach a pulse rate of 70%—in accordance with Ratey [31]—with the purpose of improving both academic goals as well as physical and mental health.

The empirical data for this article consist of eight semi-structured group interviews. Four interviews were carried out at each school. The interviewed students were randomly chosen by school staff. The only criterion was that there should be a balanced representation of girls and boys, with three girls and three boys in each group. Verbal and written consent to partake in the interviews were obtained from the participants’ parents as well as from the participating students themselves. Dates for the interviews are listed in Appendix A.

The semi-structured interviews were carried out based on a few cross-cutting issues that served as a basis for supplementary and further discussion. The cross-cutting issues were first and foremost related to the students’ attitudes toward and experiences of the ECPAs, focusing on issues such as positive and negative aspects, impact on school, health, inclusion and voluntariness. We used open questions, such as “tell me about what your expectations of ECPA were, how you have experienced ECPA, the positive and negative aspects of ECPA and its impact on health and school”. The participants had the opportunity to answer all questions and they were given time, making sure completion was achieved. Most of the interviews exhibited similar answers and attitudes. Finally, the interviews were transcribed with the purpose of analyzing and coding the interviews. A qualitative content analysis was used for the analysis of the transcribed material [32]. The present investigation was conducted in accordance with the Declaration of Helsinki for human studies and was approved for retrospective and prospective data by the Swedish Ethical Review Authority (Dnr 2019-05634).

## 3. Results

Three main themes, linked to the aim of the study, were found in the transcription analysis: (1) attitudes, (2) structural conditions and (3) impact on school and health. The results below are structured around these three themes. 

### 3.1. Attitudes

In general, the interviewed students at both schools expressed positive attitudes towards ECPAs, on a general level emphasizing a felt correlation between physical activities and health. Nevertheless, there are different reasons for their attitudes being positive. There are also those who, for various reasons, display a critical approach to ECPAs. One evident result, which concerns both schools, is that girls have a more positive attitude towards ECPAs than boys, in particular at S1. Another result is that there exist several differences between the students at S1 and S2 in terms of their views on ECPAs. There is a bundle of factors that explain why these differences appear. One prominent factor is that the students interviewed at S2, as opposed to those at S1, all like sports activities. One explanation for this could be that many students applied to S2 since ECPAs were a part of that school’s schedule, whereas ECPAs at S1 were implemented for a limited time without asking the students. The age difference may also explain the dissimilarities, since younger children in general have a more positive attitude towards school. To summarize, the younger students at S2 have generally more positive attitudes toward having ECPAs in comparison to the students at S1, who display a more varied opinion on ECPAs.

Most students at S2 agree on several issues. Not only do they think ECPAs are a welcome and stimulating break from traditional classes, but they also feel that ECPAs among other things create energy, improve concentration capacity for upcoming lessons and that schoolwork in general benefits from ECPAs.

Yes, it it’s like, after the pulse pass, after having, what’s it called, made quite an effort, you’ll focus much better during the lessons.

(Student, S2)

On the days with pulse exercise, you’re a little happier and active and the following lesson will be fun. It’s fun and you feel happy, sort of.

(Student, S2)

For example, you’re supposed to have a test, you have time to think about something else, and you kind of relax even though you are working out.

(Student, S1)

Yes, you will kind of, perhaps you’ll concentrate better if you get rid of your energy a bit, that you don’t have excess energy. Also, you might improve your fitness as well.

(Student, S1)

There are, however, also some students at S2 who point out somewhat unexpected positive aspects of ECPAs, which are not connected to possible positive effects on academic achievement. Instead, they perceive ECPAs as an acceptable way of not having to focus on more traditional subjects such as social sciences and Swedish. 

Some students at S1 have a different experience. They emphasize that they have acquired a more favorable attitude toward school since the implementation of ECPAs. They state that, thanks to ECPAs, the motivation to go to school has increased, claiming that this also has had a positive effect on their learning situation and their willingness to study.

Good for your health. You know, that’s what I believe, it is a good thing having twenty minutes every day where you work out or have physical education. It’s also good for your health.

(Student, S1)

You will be too tired to do anything. You know, it’s this kick-start in the morning, that it’s like an energy boost. If you take that away, the whole day will be longwinded and slow.

(Student, S1)

At S1 as well as at S2—in the latter case, to a larger extent—a great number of students participate in sport activities out of school—be it on their own or in a local sports club—and stress the value of sports in their lives. Most of these students welcome ECPAs both as a much-appreciated practice at school and as complementary to sports activities out of school. There is, however, no apparent correlation between sports activities out of school and a positive attitude towards ECPAs. On the contrary, a few students who practice sport nearly every day are somewhat skeptical about ECPA for two essential reasons. The most prominent one is that they do not understand the benefits of ECPAs, since they exercise sufficiently out of school.

Nothing has happened, it doesn’t matter, I practice ice-hockey four times a week, and 20 min won’t make a difference.

(Student S2)

Well, I have played basketball since before we even had ECPA and I haven’t noticed much of a difference. At the moment I have like three times…or I practice basketball three times a week so neither I think that twenty minutes make a difference.

(Student S2)

I haven’t noticed a big difference since I exercise in my spare time and all, so there is no great change in my life because I exercise a bit more in school.

(Student S1)

Furthermore, a couple of these students at S1 expressed mild criticism about ECPAs because they are mandatory and have been forced on them without their consent. They suggest the resources assigned to ECPAs could have been used for English, Swedish or mathematics. At S1, students expressed that they were stressed out, since teachers, contrary to the purpose of ECPAs, used pulse activities as an opportunity for grading the students in physical education.

Yes, it means that you fight more for the pulse [activities]. Since it was only like play time before, it might have been that you, like, don’t participate really, since you don’t think it’s worthwhile. But now when they say that it affects your grade, you do…you do participate to a larger extent.

(Student S1)

There are not many students at S2 who either express a critical approach or voice a disapproval of ECPAs. There is only one exception. Two boys believed that the 30 min twice a week was not enough time spent on exercises, a statement that could be interpreted as a rather positive approach to ECPAs and toward a school where ECPAs are an integral part.

They are quite short, and you don’t have much time, which means that many cheat and what not.

(Student S2)

At S1, similar thoughts were expressed. Some of the interviewed boys and girls claimed that the activities were either “too soft” or ineffective, in the understanding that it was impossible to reach the stipulated pulse rate of 70%. These were minor objections from students with a positive attitude.

That it would get your heart rate up, that it would be more rewarding. It’s not very rewarding. Sometimes we just play a game you know, dodge ball. It doesn’t get your heart rate up very much, it’s not very rewarding.

(Student S1)

Yes, you know, I am quite fit from the outset, but I don’t think that my fitness has improved. Since there are many workouts that are more like a walk or basketball shots. You know, you do not really improve your fitness.

(Student S1)

Yes, then you really want, at least do I feel that when we have the heart rate straps on, we’ve had them on some occasion in physical education. Then you want to work harder. It’s fun to see that your heart rate increases.

(Student S1)

Finally, there is one group in each class at S1 that seems to disapprove of ECPAs with the explanation that physical activity “is boring”, as one student laconically explains it. Most of these students are boys, and the common denominator is that they neither exercise out of school nor in school and, according to their schoolmates, manifest a negative approach to school in general. During ECPAs, their participation ranges from observing their schoolmates to a short stroll in the vicinity of the school.

As mentioned above, most students experience ECPAs as a positive, worthwhile practice that contributes to a sense of wellbeing in and out of school. Most of them underline what they experience as a nice variety of different activities such as ball games, dodgeball, circuit training, relays, Tabata and boxing.

### 3.2. Structural Conditions

Nevertheless, the students express some critical comments on aspects of ECPAs that do not directly lessen their overall positive attitudes. This could be interpreted as constructive criticism, which boils down to different aspects of how ECPAs are being carried out, on the one hand, and factors having to do with resources such as time and facilities on the other. Thus, even though the students enjoy having ECPAs, they also put forward constructive ideas about how to improve the activities.

At both schools, students ask for increasing influence on the contents of ECPAs to make the activities even more varied and more in accordance with their own wishes and on their own terms. Several students—mostly girls at S2—are critical of the competitive elements of ECPAs, something, they claim, leads to a lot of unnecessary quarrelling.

There is usually an argument between the two different teams so there is an argument after the pulse activities.

(Student S2)

You get fed up with them and that you can’t go on without having discussions and people getting angry with each other.

(Student S2)

I don’t know what it is like in the other classes, but our classes are quite competitive, at least the boys. We play dodge ball often, and there is a lot of arguing about who has won and if you have been hit, resulting in a lot of nonsense.

(Student S1)

You should simply have made a schedule before. It is a good thing, since everyone has different ideas about games and heart-rate-increasing activities. If you have the same teacher, it is easy that there will be the same exercises every time. If you had sat down and made a schedule it wouldn’t matter which teacher you would have.

(Student S1)

On the other hand, the students at S1 seem to appreciate a certain amount of competition, but on the condition that it supports their own individual performance. For example, the students at S1 very much valued the heart rate straps they initially had when ECPAs were implemented, since the device offered them an opportunity to monitor their heart rate and to maximize the benefits of the ECPAs.

There are, moreover, other problematic aspects of ECPAs which the students point out, problems not related to the actual activities, but rather to issues connected to each school’s structural conditions. One such issue involves hygiene facilities. Some of the girls at S2 complain about unhygienic conditions, because they do not have the opportunity to shower after ECPAs. A related problem is expressed by girls at S1 who either complain that some of their schoolmates refuse to shower—for reasons of insufficient privacy—or a schedule that does not allow enough time for taking a shower, with the result that they must spend the rest of the school day “sweaty and smelly”. These problems are connected to structural conditions that both schools have taken into consideration.

### 3.3. Impact on School and Health

Quite a few of the interviewed students at S1 as well as at S2 state that there is not sufficient time between ECPAs and the following lessons, let alone for having a shower. What the students see as too tight a schedule, which leads to a great deal of stress among them, is to some extent exacerbated at S1 by teachers who—according to the students—are not particularly supportive of the existence of ECPAs.

That we will be late to the following classes sometimes since it takes too much time. There is no time for having a shower afterwards and you don’t want to smell sweat.

(Student S1)

Yes, some teachers get very irritated. There are some teachers understand, but they must report late arrival when you are late. But some teachers skip that sometimes.

(Student S1)

The stress creates less motivation for ECPAs and is, according to the interviewed students, counterproductive to the idea of ECPAs with their focus on better conditions for learning and health. Thus, many of the students suggest more time between ECPAs and the following classes, and to some extent a wider acceptance of ECPAs among teachers to reduce stress, but also shower facilities at S2 and facilities suited to the students’ privacy needs at S1 to improve the matters of hygiene.

I don’t think that that many in our class have a shower since there is not much time.

(Student S1)

Because one doesn’t have time to change, I think that many don’t bother to do so.

(Student S1)

It’s because, you know, we don’t change for physical education, no pulse activities, and perhaps one has a sweaty smell and doesn’t feel very fresh during the day.

(Student S2)

Regarding the positive effects of ECPAs on teaching and learning, the interviewed students in general stress some phenomena more than others. One conspicuous feature is that most students at both schools feel that their ability to concentrate has been improved thanks to ECPAs in the morning. However, this is an effect that has a limited range of time, lasting until lunchtime. Another positive outcome that above all S2 students and a few students at S1 mention, is an enhanced sense of happiness and/or wellbeing, which in turn makes it easier and more inspiring to deal with schoolwork in general. A few students at S1 also claim that ECPAs as the first thing in the morning helps them to reduce stress in general and in examination situations in particular. Although none of the interviewed students suggest a relation between ECPAs and improved results in terms of academic achievement, there seems to be a lot of positive outcomes and benefits from ECPAs in terms of health and wellbeing, phenomena that indirectly could improve academic achievement.

When we finish at half past two, it feels good to have a pulse activity since one becomes more active and gets more energy.

(Student S2)

I think it helps, one gets more, you know, focused during the next lesson, one feels better and activated after having pulse activities. Before, if you had a long day without any physical activities, it got longwinded and boring. When you have a pulse activity you get a little bit more boosted, and it gets easier to focus.

(Student S2)

Well, it’s like what the other say, one gets more alert thanks to pulse activities. I also believe that you think school is a little better as well. Like, if you are that tired, then you think that school is hard and boring. But if you have pulse activities you become awake and think it’s fun, so you are a bit more motivated to do new things and to keep working in school. I really think it is worthwhile and it’s not only the class directly afterwards but the whole day like.

(Student S2)

One gets more concentrated and more alert.

(Student S1)

Yes, believe so. One gets the brain working in a different way than if you go straight from home to class.

(Student S1)

I think that you get more energy out of it.

(Student S1)

Furthermore, quite a few students at both schools say that they have improved their fitness since ECPAs started.

That you notice a difference, you can concentrate better and so on. I become a better swimmer as well since I exercise which makes you faster.

(Student S1)

Sure, I absolutely believe I have done that. It’s never a bad thing to exercise, really. So, several physical exercises a week—no matter what sport it is—improves fitness a bit.

(Student S1)

I’ve become much faster. I ran faster than my mother.

(Student S2, year 5)

Nonetheless, there are also students who claim that ECPAs have no impact on school or health. Most of these students belong to one of two different categories. The first category consists mainly of boys at S1 who rarely participate in ECPAs, which means that a correlation between ECPAs and school health for them does not exist. The other category consists of those who exercise almost every day out of school, and therefore claim that ECPAs are not worthwhile.

It’s not rewarding, it doesn’t help. It’s 20 min and ten minutes consist of words and that’s it.

(Student S2)

There are only two phenomena on the negative side concerning the impact of ECPAs on school and health that are brought forward by the students. The first one deals with the competitive squabble during ECPAs mentioned by the students at S2. They suggest this is a problem students bring with them from ECPAs into the classroom, resulting in further arguments during the next lesson. A similar problem, addressed by students at S1 with a positive opinion on ECPAs and their impact on the study situation, is that students at times have too much energy after engaging in ECPAs, leading to a somewhat chaotic study situation in the classroom.

You get tired of them, that you can’t like to keep going without having discussions and people getting angry with each other.

(Student S2)

Well, I think like this, you know when there is an argument like, then there is a bad atmosphere for example during the next lesson, you continue having an argument afterwards and the atmosphere is bad somehow.

(Student S2)

You know sometimes…if an overview and instruction is supposed to be ten minutes, it goes on for twenty minutes because the teacher needs to discipline the students due to the unruly situation.

(Student S1)

## 4. Discussion

The interviews with the students at both schools show that they feel that there are some major benefits of ECPAs. One important aspect, which to some extent permeates the answers, is a general positive attitude toward ECPAs among the majority when it comes to certain aspects of school and health. This is for various reasons. The most prominent ones are improved concentration abilities, reduced stress and—to a lesser extent—enhanced cognitive skills in classes directly or shortly after ECPAs. The correlation between ECPAs and higher grades, however, is all but absent in their answers. Another positive consequence expressed by the older students at S1 is that the implementation of ECPAs has led to an increased motivation not only for going to school but also for studies. Yet, the most obvious result is the fact that most students claim that ECPAs improve their health and sense of wellbeing even though the opposite can also be heard.

### Implications for School Health

The interviews reveal structural conditions as very important for ECPAs if positive implications for health are to be achieved. Although most of the interviewees are positive, they still discuss perceived shortcomings in terms of inadequate facilities, time pressure, unhygienic conditions, blurry boundaries between ECPAs and PE, uncomprehending teachers, too much focus on competition and a lack of variation, causing unnecessary stress and anxiety. To avoid risking negative effects of ECPAs on health issues such as stress—which other studies also have identified [33,34]– it is of uttermost importance that schools provide basic structural conditions for ECPAs. Like other studies [3,4,5,6,25,28,29,31], our results also suggest that it is imperative that both students and teachers be involved in the activities, that school personnel are educated and that motivation and lust are all necessary conditions for ECPAs. Hence, the importance of including the students’ perspectives in the planning and implementation of ECPAs with the objective of improved health.

The answers indicate that ECPAs can be one way of stimulating health-promoting work in school. However, there are some issues that need to be discussed from the vantage point of our study’s results. First, there is the question of how to approach those students who—for various reasons—have a negative attitude toward ECPAs. From a health-promoting perspective, this problem mainly concerns students who neither take part in ECPAs nor in any physical activities out of school. This category of students is arguably the one in most need of increasing their daily level of physical activities. Thus, one key issue for schools is how to find ways of motivating them to embrace physical activities and to avoid reinforcing already existing sedentary habits. If schools are to contribute to the reduction in present and future health problems due to non-physical habits among children and teenagers, a phenomenon that researchers have warned about [1,6,26,27,35,36,37], it is imperative to find methods of including and motivating this category of students in physical activities such as ECPAs. 

One such method, expressed by the interviewed students, is to include the students in the ECPAs, both in terms of content and form. Involving the students—and the concerned teachers and other school personnel—could be a way of creating a more inclusive and democratic practice in which issues such as competition, stress, hygiene and individual approaches to ECPAs could be discussed to meet the needs of each student, even the most recalcitrant ones. 

Second, the problem with students who do not exercise in or out of school cannot be discussed without taking age and gender differences into consideration. There is a substantial decrease in physical activities among students on their way from childhood to adolescence [36,38]. This pattern may be one explanation as to why there are no students at S2 who do not participate in or express negative attitudes towards ECPAs like one group at S1. Not only do these students not perform any physical activities, but they also articulate an unwillingness to participate in ECPAs or any other physical activities, which makes the issue of motivation and inclusion even more challenging from a health perspective, let alone from the perspective of preparing children for an active life, which increases the chances of a physical and active life in the long term [1,7,17,39]. This shows the importance of encouraging and enabling adolescents to participate in vigorous exercising to promote positive mental health [30,35]. Our results demonstrate that this is especially important for teenage students who do not want to participate in any form of physical activity.

Furthermore, our results indicate a difference in gender. Those not interested in physical activities in or out of school at S1 are mainly boys. The results from S1 show that girls are more active in ECPAs than boys. In that sense, our study to some extent differs from earlier research that claim that girls in general, and to a larger extent, do not exercise as much as needed as compared to boys [11,30]. One explanation for this difference could be that the result of our study is limited to a school context. 

A third issue which needs to be emphasized is the specific ECPAs profile of S2, which attracts students interested in physical activities. Most students at S2 also acknowledge that they applied for the school due to its ECPAs profile. The students at S1 had no such choice, since ECPAs were a time-limited project implemented after they had applied for that school. Thus, if a school presents itself with an ECPAs profile, it will probably attract students—independently of age—with a positive attitude toward sports, whereas students who do not take part in any physical activities will apply for schools without such a profile. This could lead to a consolidation or even an increase in children and youngsters who do not reach the recommended level of daily physical activities, with possible negative consequences for future school health issues. 

This leads us to the last question that the study raises: What responsibility does school as an institution—and in the long run, society—have and what role should it play concerning students’ physical activities and their health—present and future? 

The most important outcome of this study is the emphasis on students and their experiences and perspectives of ECPAs. By including the students, it has been possible to identify the opportunities and challenges of ECPAs, concerning issues such as attitudes, structural conditions and impact on school and health.

However, since the study is rather limited in scope, further studies with both longitudinal designs and additional methods, such as quantitative data and mixed methods, are requested to gain more knowledge not only of students’ attitudes and felt experiences, but also on measurable outcomes of ECPAs in terms of health over the short-to-medium and long term. Furthermore, issues such as the role and responsibility of school and how to implement ECPAs in a both including and motivating way need to be addressed more thoroughly.

## 5. Conclusions

To conclude, the interviews show that students feel that ECPAs have a positive impact on their health, and to some extent on school-related issues such as concentration and reduced stress. Still, it is important to clearly distinguish ECPAs from PE to enable the students to focus on pulse activities without having to worry about grades. In the long term, ECPAs may contribute to better physical habits among children and adolescents as well as improved health and an awareness of the benefits of a healthy lifestyle.

## Data Availability

The dataset supporting the conclusion of this article is available from the authors upon reasonable request and the completion of a data transfer agreement.

## Appendix A

Interviews

Group interview 1 at school 1, 25 February 2020

Group interview 2 at school 1, 25 February 2020

Group interview 3 at school 1, 13 March 2020

Group interview 4 at school 1, 13 March 2020

Group interview 5 at school 2, 25 May 2020

Group interview 6 at school 2, 25 May 2020

Group interview 7 at school 2, 25 May 2020

Group interview 8 at school 2, 25 May 2020

## References

[1] Mayer D.G., Faigenbaum A.D., Stracciolini A., Hewett T.E., Micheli L.J., Best T.M. (2013). Comprehensive management strategies for physical inactivity in youth. Curr. Sport Med. Rep..

[2] International Society for Physical Activity and Health (2021). Infographic. ISPAH’s eight investments that work for physical activity: Infographic, animation and call to action. Br. J. Sport. Med..

[3] Boržíková I., ChovanovÁ E., Majherová M., Kandráč R. (2020). Development of basic motor competencies among standard population during the prepubertal period. J. Phys. Educ. Sport.

[4] Hyde E.T., Gazmararian J.A., Barrett-Williams S.L., Kay C.M. (2020). Health Empowers You: Impact of a School-Based Physical Activity Program in Elementary School Students, Georgia, 2015–2016. J. Sch. Health.

[5] Volmut T., Šimunič B. (2021). Effect of unstructured 15-minute active recess on children’s daily physical activity. J. Phys. Educ. Sport.

[6] Øien A.M., Solheim I.J. (2019). Physical activity and interaction for less active pupils at elementary school: The experiences of teachers and parents. Eur. Phys. Educ. Rev..

[7] Fritz J. (2017). Physical Activity During Growth. Effects on Bone, Muscle, Fracture Risk and Academic Performance. Ph.D. Thesis.

[8] Bangsbo J., Krustrup P., Duda J., Hillman C., Andersen L.B., Weiss M., Williams C.A., Lintunen T., Green K., Hansen P.R. (2016). The Copenhagen consensus Conference 2016: Children, Youth and Physical Activity in schools and during leisure time. Br. J. Sports Med..

[9] Walker T.J., Craig D.W., Pavlovic A., Thiele S., Kohl H.W. (2020). Associations between gender, school socioeconomic status, and cardiorespiratory fitness among elementary and middle school students. BMC Public Health.

[10] Giroir B.P., Wright D. (2018). Physical activity guidelines for health and prosperity in the United States. JAMA.

[11] Nyberg G. (2017). Få unga rör sig Tillräckligt. Rapport.

[12] Hollis J., Sutherland R., Williams A., Campbell E., Nathan N., Wolfenden L. (2017). A systematic review and meta-analysis of moderate-to-vigorous physical activity levels in secondary school physical education lessons. Int. J. Behav. Nutr. Phys. Act..

[13] Brusseau T., Hannon J., Burns R. (2016). The effect of a comprehensive school physical activity program on physical activity and health-related fitness in children from low-income families. J. Phys. Act. Health.

[14] World Health Organization (WHO) Global Recommendations on Physical Activity for Health 2010. https://www.who.int/news-room/fact-sheets/detail/physical-activity.

[15] Kahlin Y., Werner S., Alricsson M. (2014). A physical activity program for Swedish physically inactive female high school students: A controlled intervention study. J. Phys. Act Health.

[16] Kahlin Y., Werner S., Edman G., Raustorp A., Alricsson M. (2016). Self-esteem and personality traits in Swedish physically inactive female high school students. A controlled intervention study. Int. J. Adolesc. Med. Health.

[17] Ericsson I., Karlsson M.K. (2014). Motor skills and school performance in children with daily physical education in school–a 9-year intervention study. Scand. J. Med. Sci. Sport..

[18] Bunketorp Käll L., Malmgren H., Olsson E., Lindén T., Nilsson M. (2015). Effects of a curricular physical activity intervention on children´s school performance, wellness, and brain development. J. Sch. Health.

[19] Brusseau T.A., Hannon J.C. (2015). Impacting children’s health and academic performance through comprehensive school physical activity programming. Int. Electron. J. Elem. Edu..

[20] Ericsson I., Cederberg M. (2015). Physical activity and school performance: A survey among students not qualified for upper secondary school. Phys. Educ. Sport Pedagog..

[21] Donnelly J.E., Hillman C.H., Castelli D., Etnier J.L., Lee S., Tomporowski P., Lambourne K., Szabo-Reed A.N. (2016). Physical Activity, Fitness, Cognitive Function and Academic Achievement in Children: A Systematic Review. Med. Sci. Sport Exerc..

[22] Andersen M.P., Mortensen R.N., Vardinghus-Nielsen H., Franch J., Torp-Pedersen C., Bogglid H. (2016). Association between physical fitness and academic achievement in a cohort of Danish school pupils. J. Sch. Health.

[23] Cöster M., Fritz J., Karlsson C., Rosengren B., Karlsson M. (2018). Extended physical education in children aged 6–15 years was associated with improved academic achievement in boys. Acta Paediatr..

[24] De Greeff J., Bosker R., Oosterlaan J., Visscher C., Hartman E. (2018). Effects of physical activity on executive functions, attention and academic performance in preadolescent children: A meta-analysis. J. Sci. Med. Sport.

[25] Ericsson I. (2020). Physical activity interventions in school and their impact on scholastic performance. Res. Inves. Sport. Med..

[26] Rowland T.W. (2007). Promoting physical activity for children’s health: Rationale and strategies. Sports Med..

[27] Faigenbaum A.D., Kraemer W.J., Blimkie C.J.R., Jeffereys I., Micheli L.J., Nikta M., Rowland T.W. (2009). Youth resistance training: Updated position statement paper from the national strength and conditioning association. J. Strength Cond. Res..

[28] Lindgren E.-C., Haraldsson K., Håman L. (2019). Voices from pupil participation in the health promotion intervention “Pulse for Learning and Health [PuLH]” in Primary and Middle School. Int. J. Environ. Res. Public Health.

[29] Seger I., Lundvall S., Eklund A., Jamshidpey A., Takats J., Stålman C., Tidén A., Andersson E.A. (2022). A Sustainable Swedish School Intervention with Extra Aerobic Exercise—Its Organization and Effects on Physical Fitness and Academic Achievement. Sustainability.

[30] Molcho M., Gavin A., Goodwin D. (2021). Levels of Physical Activity and Mental Health in Adolescents in Ireland. Int. J. Environ. Res. Public Health.

[31] Ratey J.J., Hagerman E. (2008). Spark–The Revolutionary New Science of Exercise and the Brain.

[32] Creswell J.W. (2014). Research Design. Qualitative, Quantitative and Mixed Methods Approaches.

[33] Resaland G.K., Aadland E., Moe V.F., Aadland K.N., Skrede T., Stavnsbo M., Suominen L., Steene-Johannessen J., Glosvik O., Andersen J.R. (2016). Effects of physical activity on schoolchildren’s academic performance: The Active Smarter Kids (ASK) cluster-randomized controlled trial. Prev. Med..

[34] Sjöwall D., Hertz M., Klingberg T. (2017). No long-term effect of physical activity intervention on working memory or arithmetic in preadolescents. Front Psychol..

[35] Batista M., Ramos L., Santos J., Serrano J., Petrica J., Honório S. (2022). Profile of self-concept and self-esteem on the academic performance among practitioners of physical education and extracurricular activities in middle-school students. Minerva Pediatr..

[36] Pan Y., Zhou D., Shek D.T. (2022). After-school extracurricular activities participation and depressive symptoms in Chinese early adolescents: Moderating effect of gender and family economic status. Int. J. Environ. Res. Public Health.

[37] Pan Y., Zhou D., Shek D.T.L. (2022). Participation in After-School Extracurricular Activities and Cognitive Ability Among Early Adolescents in China: Moderating Effects of Gender and Family Economic Status. Front. Pediatr..

[38] Faskunger J. (2013). Fysisk Aktivitet Och Folkhälsa.

[39] Kanda D., Walker A., Siekirk N., Colquitt G. (2021). The Relationship Between Physical Activity and School Success Among Children With and Without Special Health Care Needs. J. Sch. Health.

